# Carotid body potentiation during chronic intermittent hypoxia: implication for hypertension

**DOI:** 10.3389/fphys.2014.00434

**Published:** 2014-11-12

**Authors:** Rodrigo Del Rio, Esteban A. Moya, Rodrigo Iturriaga

**Affiliations:** ^1^Laboratorio de Neurobiología, Departamento Fisiología, Facultad de Ciencias Biológicas, Pontificia Universidad Católica de ChileSantiago, Chile; ^2^Laboratory of Cardiorespiratory Control, Center of Biomedical Research, Universidad Autónoma de ChileSantiago, Chile

**Keywords:** carotid body, intermittent hypoxia, autonomic imbalance, hypertension

## Abstract

Autonomic dysfunction is involved in the development of hypertension in humans with obstructive sleep apnea, and animals exposed to chronic intermittent hypoxia (CIH). It has been proposed that a crucial step in the development of the hypertension is the potentiation of the carotid body (CB) chemosensory responses to hypoxia, but the temporal progression of the CB chemosensory, autonomic and hypertensive changes induced by CIH are not known. We tested the hypothesis that CB potentiation precedes the autonomic imbalance and the hypertension in rats exposed to CIH. Thus, we studied the changes in CB chemosensory and ventilatory responsiveness to hypoxia, the spontaneous baroreflex sensitivity (BRS), heart rate variability (HRV) and arterial blood pressure in pentobarbital anesthetized rats exposed to CIH for 7, 14, and 21 days. After 7 days of CIH, CB chemosensory and ventilatory responses to hypoxia were enhanced, while BRS was significantly reduced by 2-fold in CIH-rats compared to sham-rats. These alterations persisted until 21 days of CIH. After 14 days, CIH shifted the HRV power spectra suggesting a predominance of sympathetic over parasympathetic tone. In contrast, hypertension was found after 21 days of CIH. Concomitant changes between the gain of spectral HRV, BRS, and ventilatory hypoxic chemoreflex showed that the CIH-induced BRS attenuation preceded the HRV changes. CIH induced a simultaneous decrease of the BRS gain along with an increase of the hypoxic ventilatory gain. Present results show that CIH-induced persistent hypertension was preceded by early changes in CB chemosensory control of cardiorespiratory and autonomic function.

## Introduction

Obstructive sleep apnea (OSA), a highly prevalent sleep-breathing disorder is recognized as an independent risk factor for systemic diurnal hypertension (Gozal and Kheirandish-Gozal, 2008; Somers et al., 2008; Dempsey et al., 2010). Repeated episodes of partial or complete obstruction of the upper airways in OSA leads to intermittent hypoxia and hypercapnia, sleep fragmentation and arousal. The resulting hypoxia and hypercapnia stimulate the carotid body (CB) chemoreceptor's eliciting ventilatory, sympathetic and cardiovascular reflex responses. Remarkably, among all the disturbances produced by OSA, the chronic intermittent hypoxia (CIH) is considered the main factor for the development of hypertension (Gozal and Kheirandish-Gozal, 2008; Somers et al., 2008; Dempsey et al., 2010). Although the link between OSA and diurnal persistent hypertension is well established, the mechanisms responsible for the hypertension are not entirely known. Oxidative stress, inflammation and increased sympathetic outflow have been proposed to mediate the OSA-induced hypertension (Lavie, 2003; Gozal and Kheirandish-Gozal, 2008; Somers et al., 2008). The pathogenic mechanisms involved in the development of hypertension have been extensively studied in animal models of CIH, which simulate the hypoxic-reoxygenation cycles observed in OSA patients and reproduce the main cardiovascular OSA features (Fletcher et al., 1992; Prabhakar et al., 2005; Iturriaga et al., 2009; Pack and Gislason, 2009).

Studies performed by Fletcher et al. (1992) showed that the denervation of the CBs prevents the development of hypertension in rats exposed to CIH. Despite the findings reported by Fletcher et al. (1992) suggesting the contribution of the CB in the CIH-induced hypertension, the proposal that the peripheral CB chemoreceptor plays a key role in the progression of the hypertension did not receive much attention. However, a growing body of new evidence suggests that an enhanced CB chemosensory afferent responsiveness to hypoxia contributes to the OSA-induced hypertension (Peng et al., 2003; Rey et al., 2004; Dick et al., 2007; Smith and Pacchia, 2007; Weiss et al., 2007; Iturriaga et al., 2009; Marcus et al., 2010; Prabhakar and Semenza, 2012). Indeed, enhanced sympathetic and cardiorespiratory responses to acute hypoxia in OSA patients have been attributed to a heightened CB chemoreflex drive (Narkiewicz et al., 1998a, 1999; Lusina et al., 2006). In addition, studies performed in animals exposed to CIH have shown that CIH selectively enhances CB chemosensory and ventilatory responses to hypoxia (Peng et al., 2003; Peng and Prabhakar, 2004; Rey et al., 2004; Iturriaga et al., 2009; Del Rio et al., 2010) and produces long-term facilitation of motor ventilatory activity (Mitchell et al., 2001; McGuire et al., 2003). Recordings from the carotid sinus nerve confirmed that CIH enhances chemosensory discharge evoked by acute hypoxia (Peng et al., 2003; Peng and Prabhakar, 2004; Rey et al., 2008; Del Rio et al., 2010). Moreover, Peng et al. (2003) found that the CB discharge in normoxia was higher in rats exposed to CIH, applied during 8 h for 10 days. Thus, CIH increases CB chemosensory discharge in normoxia and enhanced the responses to hypoxia. In addition to the potentiation of the CB chemosensory response, CIH induces plasticity of the CB chemosensory activity manifested as long-term facilitation (LTF). Indeed, Peng et al. (2003) reported that chemosensory excitation can be induced by repetitive acute intermittent hypoxia when animals were pre-conditioned using CIH. Indeed, they reported that following 10 episodes of 12% O_2_ for 15 s, interspersed with 5 min of 95% O_2_, the chemosensory discharge measured in anesthetized rats increased with each episode of hypoxia and returned to baseline after terminating the 10th hypoxic episode. However, when rats were previously exposed to CIH (15 s of 5% O_2_ 8 h/day) for 10 days, and then submitted to the same pattern of 10 intermittent hypoxic episodes, they observed a large increase in the baseline CB discharge, which persisted for 60 min after the end of the hypoxic stimulus.

It has been proposed that the attenuation of the cardiac baroreflex and alterations of heart rate variability (HRV) contribute to the CIH-induced hypertension in OSA patients (Shiomi et al., 1996; Narkiewicz et al., 1998a) and adult animals exposed to CIH (Rey et al., 2004, 2008; Lai et al., 2006; Sica and Zhao, 2006). In an elegant study performed by Lai et al. (2006) they showed that autonomic dysfunction in rats exposed to CIH are related to BP rises. Moreover, they found that the rats exposed to CIH display a marked decreased in baroreflex sensitivity (BRS) and HRV along with augmentation of the ventilatory responses to acute hypoxia (Lai et al., 2006). However, whether these changes were related to CB potentiation was not studied. Furthermore, there are no integrative studies showing that the CB chemosensory potentiation heads the autonomic imbalance and/or the development of hypertension induced by CIH. Therefore, we seek to unite under one study if the enhanced CB chemoreflex drive precedes the autonomic imbalance and the hypertension during CIH. Accordingly, we studied i) the progression of changes in CB afferent chemosensory activity and ventilatory reflex response in response to several inspired PO_2_ levels, ii) changes in autonomic control using indirect methods (HRV and BRS) and iii) changes in BP (measured 16–20 h after the end of the last intermittent hypoxic cycle) in rats exposed to 7, 14, and 21 of CIH. In addition, we studied the relation between the changes in HRV and BRS with the augmentation of the CB-mediated ventilatory chemoreflex gain.

## Materials and methods

### Animals

Experiments were performed on male Sprague-Dawley rats weighting initially ~200 g, fed with standard diet *ad libitum* and kept on a 12-h light/dark schedule (7:30 a.m. to 7:30 p.m.). The protocol was approved by the Bioethical Committee of the Biological Sciences Faculty, Pontificia Universidad Católica de Chile, and was performed according to the Guide for Care and Use of Laboratory Animals, published by the US National Institute of Health (NIH publication no. 85–23, revised 1996).

### Chronic intermittent hypoxia

Unrestrained, freely moving rats housed in individual chambers (12 × 35 cm, 2.2 l) were exposed to a CIH protocol consisting of hypoxic cycles of F_I_O_2_ 5% for 20 s, followed by room air for 280 s, applied 12 times/h for 8 h/day during 7, 14, and 21 days (Iturriaga et al., 2009; Del Rio et al., 2010, 2011, 2012). Chambers were equipped with a rear N_2_ inlet to produce periodic hypoxic episodes, and a front air extractor, which enables to recover the normoxic level. A computerized system controls the solenoid valve and the alternating cycles of the extractors. During hypoxic exposure, the extractors were stopped, while rear solenoid valves allow 100% N_2_ flows into the chambers. The O_2_ level in the chambers was monitored with an oxygen analyzer (Ohmeda 5120, USA). The CO_2_ in the chamber was maintained low by continuous air extraction. A group of age-matched rats was exposed to sham condition, whereas the N_2_ gas was replaced by means of flushing equal flow of compress air into the chambers. The room temperature was kept at 23–25°C and the intermittent hypoxic and sham patterns were applied from 8:30 a.m. to 4:30 p.m.

### Recordings of physiological variables

Experiments were performed 16 to 20 h after the end of the last intermittent hypoxic cycle. Rats were anesthetized with sodium pentobarbitone (40 mg/kg I.P.), a condition necessary for the study of CB afferent chemosensory discharges, followed by additional doses when necessary to maintain a level of surgical anesthesia. Rats were placed in supine position and the rectal temperature was maintained at 38.0 ± 0.5°C with a regulated heating pad. The trachea was cannulated for airflow recording, and connected to a pneumotachograph to measure tidal volume (V_T_), respiratory frequency (f_R_), and minute inspiratory volume (V_I_) as previously described (Del Rio et al., 2010, 2012). One femoral artery was cannulated with a polyethylene tube, filled with 50 IU/ml of heparin solution for measuring arterial blood pressure (BP) with a transducer (Statham P23, USA). The mean arterial blood pressure (MABP) from the BP signal. Signals were acquired with an analog-digital system PowerLAB 8SP, calibrated, and analyzed with the Chart 7.1-Pro software (ADInstruments, Australia). We measured the ventilatory responses elicited by several levels of PO_2_ (5 to 670 mmHg), maintained until the response was in a semi steady state (~15–30 s). The PO_2_ level was achieved by mixing different amounts of 100% O_2_ and 100% N_2_. The O_2_ level of each gas mixture was monitored with an oxygen analyzer (Ohmeda 5120, USA).

### Heart rate signal acquisition and processing

HRV was assessed following the guidelines from the Task Force of the European Society of Cardiology and the North American Society of Pacing and Electrophysiology (Task Force, 1996). The electrocardiogram (ECG) was measured using the II Einthoven lead. Three stainless steel 22G needle electrodes were localized in the insertion of the right (G1) and left (GND) front legs, and in the left (G2) rear leg. Accordingly, 10 min of ECG recordings were digitally acquired at 2 kHz prior to any maneuver. The ECG was examined for detection of ectopic beats that were manually deleted from the recording and interpolated. The ECG signals were analyzed with the HRV module of the Chart 6.1-Pro software. The power spectrum of R-R interval data was obtained using a Fast Fourier Transform algorithm after application of the Hann window. The spectrum of R-R intervals was assessed using the following frequency bands as previously described in rats (Lai et al., 2006; Del Rio et al., 2010): very-low frequency (VLF): DC-0.04 Hz, low frequency (LF): 0.04–0.6 Hz and high frequency (HF): 0.6–2.4 Hz. Calculations considered the relative power of the LF and HF powers expressed as normalized units and the LF/HF ratio.

### Spontaneous baroreflex sensitivity

Spontaneous BRS over heart rate was calculated using the spectral method (Pagani et al., 1988; Laude et al., 2004). Briefly, the α-coefficient index was calculated to estimate the BRS as the spectral transfer function between heart rate and blood pressure variability. BRS was calculated using the low frequency spectral components of the respective signals since it has been shown that the coupling between RR interval and SBP have a non-baroreflex origin in the high frequency range and leads to results that are not all related to BRS (Parati et al., 2000).

For the interaction analysis, BRS was calculated by the sequence method, up-sequences of four or more heart beats, where the R-R interval changed in the same direction and presented a linear correlation coefficient higher than 0.85 were included in the analysis. The average slope of the regression lines was calculated using the HemoLab software (www.haraldstauss.com) to provide the estimation of the BRS in ms/mmHg (Straus et al., 2006).

### Carotid body chemosensory recordings

The CB chemosensory discharge was measured as previously described (Iturriaga et al., 2009; Del Rio et al., 2010, 2011, 2012). Briefly, one carotid sinus nerve was dissected and placed on a pair of platinum electrodes and covered with warm mineral oil. The neural signal was pre-amplified (Grass P511, USA), filtered (30–500 Hz) and fed to an electronic spike-amplitude window discriminator allowing the selection of action potentials of given amplitude above the noise. The lower level of detection was setup when rats breathed 100% O2 (Dejours Test) and the upper level of the windows was leave open. The selected potentials were counted with a frequency meter to assess the CB chemosensory frequency of discharge (*f*_x_), expressed in Hz. Signals were acquired with an analog-digital system PowerLAB 8SP and analyzed with the Chart 7-Pro software (ADInstruments, Australia). The chemosensory discharge was measured at several levels of PO_2_ (5 to 670 mmHg), maintained until the response was in semi steady state (~10–20 s). Rats breathed spontaneously during the experiments. At the end of the experiments, rats were killed by an overdose of sodium pentobarbitone (100 mg/kg I.P.).

The CB chemosensory discharges response curves were fitted to the inspiratory PO_2_, according to the exponential function (Berkenbosch et al., 1991, 1997).

$$f_{\text{x}}=bas\;f_{\text{x}}+G\;exp(-K/PO_{2})$$

In which, *G* (max − bas) represents the overall gain, *K* is the shape parameter, and *bas* is the value of ventilation or CB chemosensory discharge measured during hyperoxic challenge. CB chemosensory frequency of discharge (*f*_x_), was expressed in Hz.

### Carotid body-mediated chemoreflex drive gain

To measure the CB afferent activity gain and the ventilatory reflex gain, the oxygen concentration dependent response curves were fitted to the inspiratory PO_2_, according to the exponential function (Berkenbosch et al., 1991, 1997).

$$V_{I}=bas\;V+G\;exp(-K/PO_{2})$$

In which, *G* (max − bas) represents the overall gain, *K* is the shape parameter, and *bas* is the value of ventilation measured during hyperoxic challenge. Inspiratory volume (*V*_I_) expresses as% of normoxia.

### Statistical analysis

The data was expressed as mean's ± *SE*. Paired comparisons between two groups were performed with the Student *T*-test, and differences between 3 or more groups were assessed with one or Two-Way ANOVA tests, followed by Newman-Keuls *post-hoc* comparisons. All analyses were done with the statistical significance set at *P* < 0.05.

## Results

### Effects of CIH on baseline physiological variables

CIH produced a significant increase in BP (*P* < 0.01) after 21 days, but not after 7 or 14 days of CIH (Table 1). Indeed, MABP measured 16–20 h after the last hypoxic episode was significantly higher (*P* < 0.001) in rats exposed to CIH for 21 days (137.8 ± 3.0 mmHg, *n* = 16) than in Sham animals (103.2 ± 3.5 mmHg, *n* = 11). No changes in heart rate (H_R_) were found among groups. Ventilatory (V_T_, FR and V_I_) measurements taken during normoxia (PO_2_ ~ 150 ± 10 mm Hg) from CIH-rats were not significantly different compared to the values obtained from Sham animals (See Table 1). However, CB chemosensory discharges measured in normoxia at the beginning of the recordings, showed a significant 1.5-fold increase in rats exposed to CIH for 7, 14, and 21 days compared to the values measured in Sham rats.

**Table 1:** **Changes in physiological variables induced by CIH in rats**.

	**Sham**	**CIH 7 days**	**CIH 14 days**	**CIH 21 days**
MABP (mmHg)	103.2 ± 3.5	97.9 ± 3.5	103.0 ± 4.2	137.8 ± 3.0^**^
H_R_ (1/min)	426.7 ± 16.9	413.6 ± 16.2	419.8 ± 16.8	402.9 ± 13.9
V_T_ (ml/kg)	4.4 ± 0.5	3.5 ± 0.3	3.1 ± 0.3	3.6 ± 0.4
f_R_ (1/min)	73.5 ± 5.6	82.4 ± 7.6	86.9 ± 10.5	73.5 ± 3.4
$\dot{V}$ (ml/min kg)	331.5 ± 48.0	280.2 ± 25.6	252.3 ± 57.9	272.9 ± 37.4
*f*_x_(Hz)	47.8 ± 5.6	71.9 ± 3.7^*^	81.7 ± 4.4^*^	80.7 ± 11.4^*^
Weight gain (g/day)	3.6 ± 0.2	2.5 ± 0.2^*^	1.8 ± 0.3^**^	2.3 ± 0.2^**^

*MABP, mean arterial blood pressure; H_R_, heart rate; V_T_, tidal volume; f_R_, respiratory rate; $\dot{V}$, minute inspiratory volume; *f*_x_, frequency of carotid chemosensory discharges measured in normoxia (PO_2_ ~ 150 ± 10 mm Hg)*.

* P < 0.05;

** *P < 0.01 CIH groups vs. Sham group; Neuman-Keuls post-hoc test after One-Way ANOVA (n = 8–12)*.

### CIH-induce early carotid body chemosensory potentiation and increase the ventilatory chemoreflex drive

**Figure 1:**
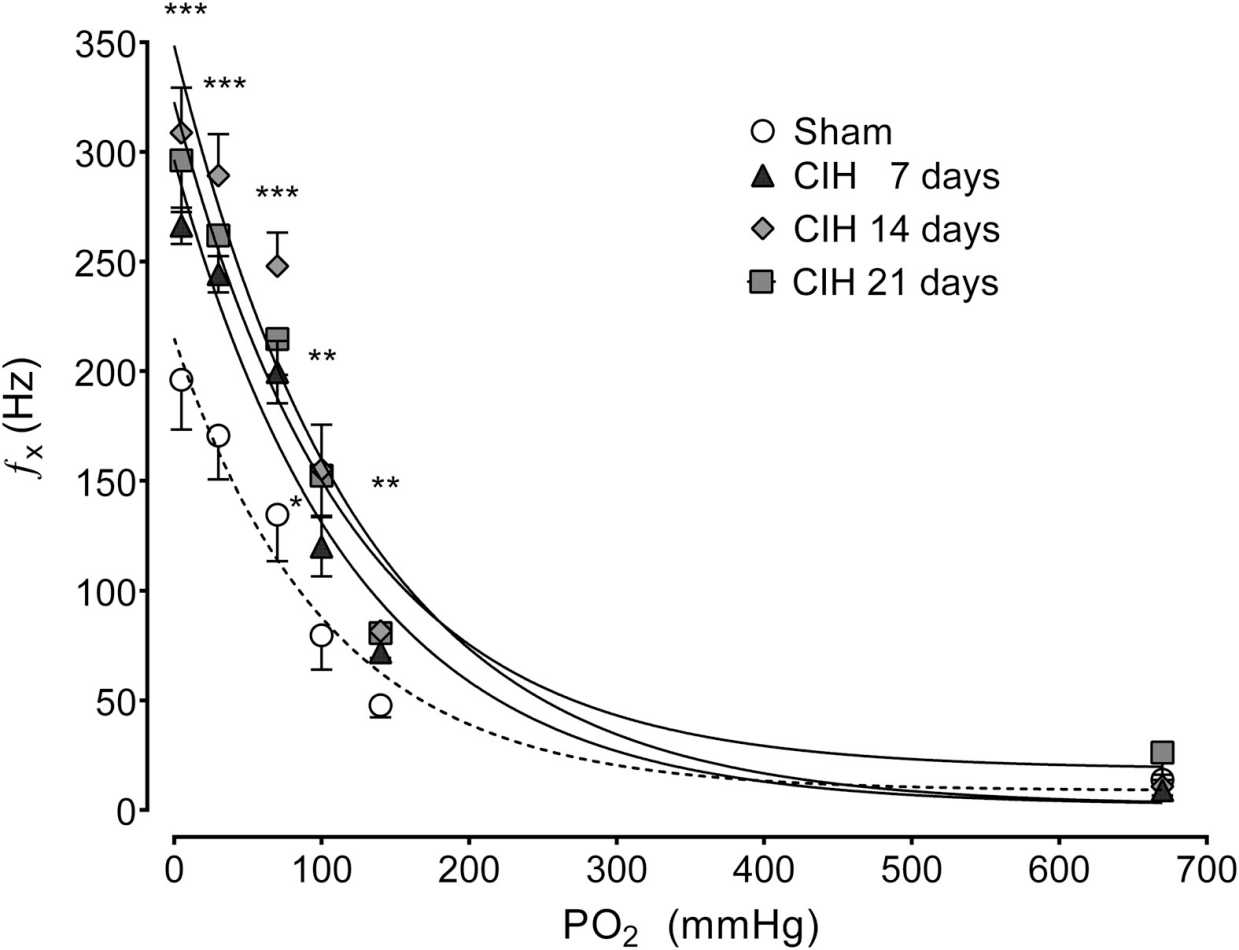
**Effects of CIH on CB chemosensory responses to several levels of inspired PO_2_**. *f*_x_, Carotid chemosensory frequency of discharge in Hz. ^***^P < 0.001, ^**^P < 0.01, ^*^P < 0.05 with sham condition. Newman-Keuls test after Two-Way ANOVA. *n* = 6–8 rats in each group. The chemosensory discharge was fitted to the inspired PO_2_ using an exponential fit [see Materials and Methods *f*_x_ = bas f_x_ + G exp(-K/PO_2_)]. The coefficients of correlation for the different curves were: Sham *r* = 0.82, CIH 7 days *r* = 0.94, CIH 14 days *r* = 0.92 and CIH 21 days *r* = 0.85. *P* < 0.01.

**Figure 2:**
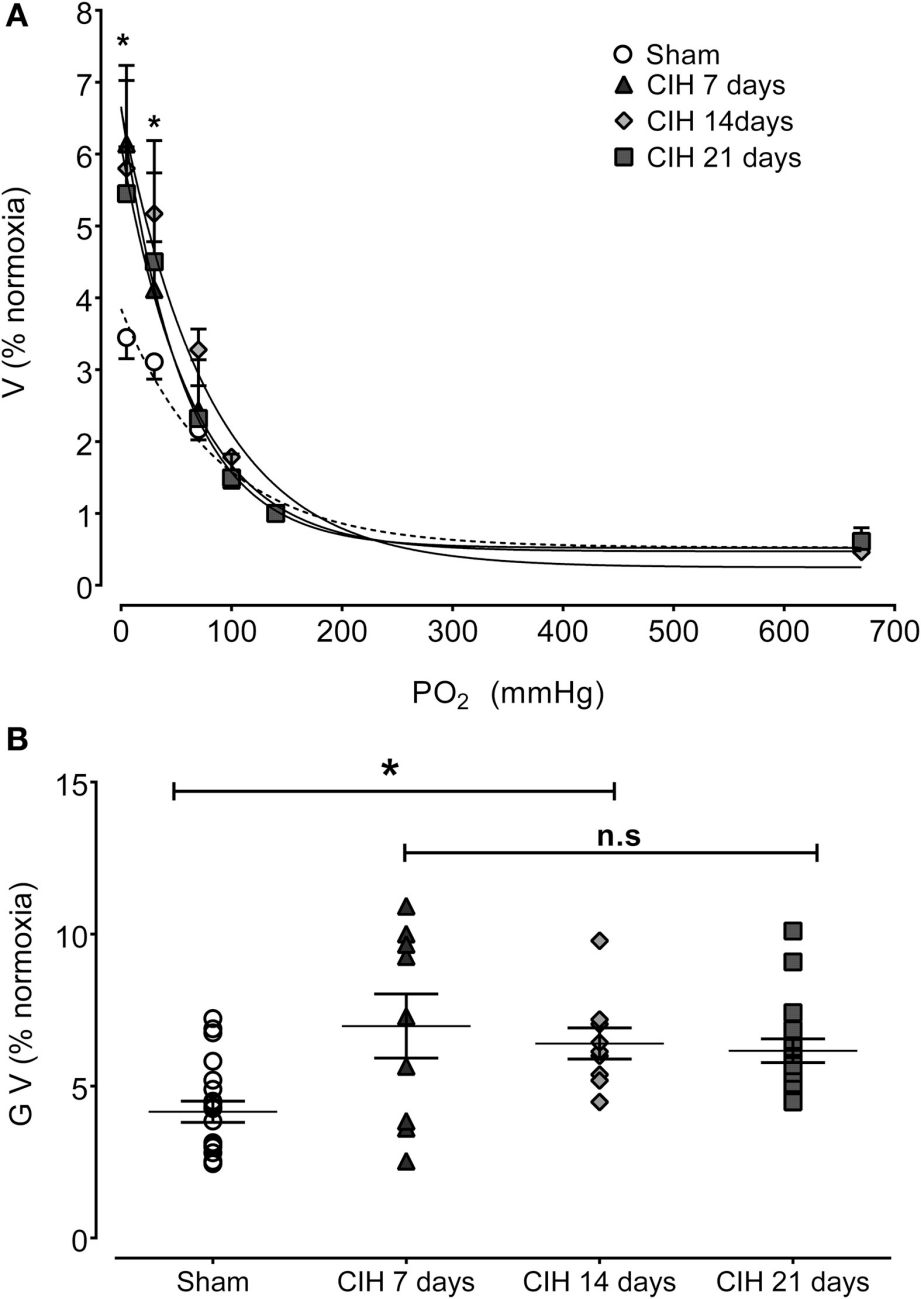
**Effects of CIH on reflex ventilatory minute volume in response to several levels of inspired PO_2_**. **(A)** Overall ventilatory curves. **(B)** Hypoxic ventilatory gain (G V as% baseline) corresponding to individual values. Ventilation expressed as percentage of normoxia ventilatory responses. ^*^P < 0.01 with sham condition. Newman-Keuls test after Two-Way ANOVA in **(A)** and One-Way ANOVA in **(B)**. *n* = 8–10 rats in each group. Ventilation was fitted to the inspired PO_2_ using an exponential fit [see Materials and Methods *V*_I_ = bas V + G exp(-K/PO_2_)]. The coefficients of correlation for the different curves were: Sham *r* = 0.82, CIH 7 days *r* = 0.77, CIH 14 days *r* = 0.91 and CIH 21 days *r* = 0.86. *P* < 0.01.

The CB chemosensory response to acute hypoxia markedly increased after 7 days of CIH, effect that persists following 14 and 21 days of CIH exposure. The effects of CIH on *f*_x_ at several PO_2_ levels (from 5 to 670 mmHg) are summarized in Figure 1. The Two-Way ANOVA analysis showed that the overall chemosensory curves for PO_2_ were different in CIH-rats exposed to 7, 14, and 21 days of CIH (*P* < 0.01) compared to the curve obtained in Sham rats. The Newman-Keuls test showed that CB chemosensory discharge was higher (*P* < 0.01) in the normoxic and hypoxic range in rats exposed to 7, 14, and 21 days of CIH. Accordingly, rats exposed to CIH displayed enhanced ventilatory responses (V_I_) to acute hypoxia. Figure 2 shows the effects of CIH on V_I_ at several inspiratory PO_2_ levels (5 to 670 mmHg). The overall curves for the relationship between V_I_ and PO_2_ were significantly different at 7, 14, and 21 days of CIH, compared with the curve obtained in Sham rats (*P* < 0.01, Two-Way ANOVA). Thus, 7 days of CIH were enough to potentiate the reflex ventilatory responses to hypoxia, effect that persisted until the day 21 of CIH exposure. Moreover, the gain of the CB-mediated ventilatory reflex response to acute hypoxia was enhanced by CIH (Figure 2B).

### CIH reduce the baroreflex sensitivity and shift heart rate variability

**Figure 3:**
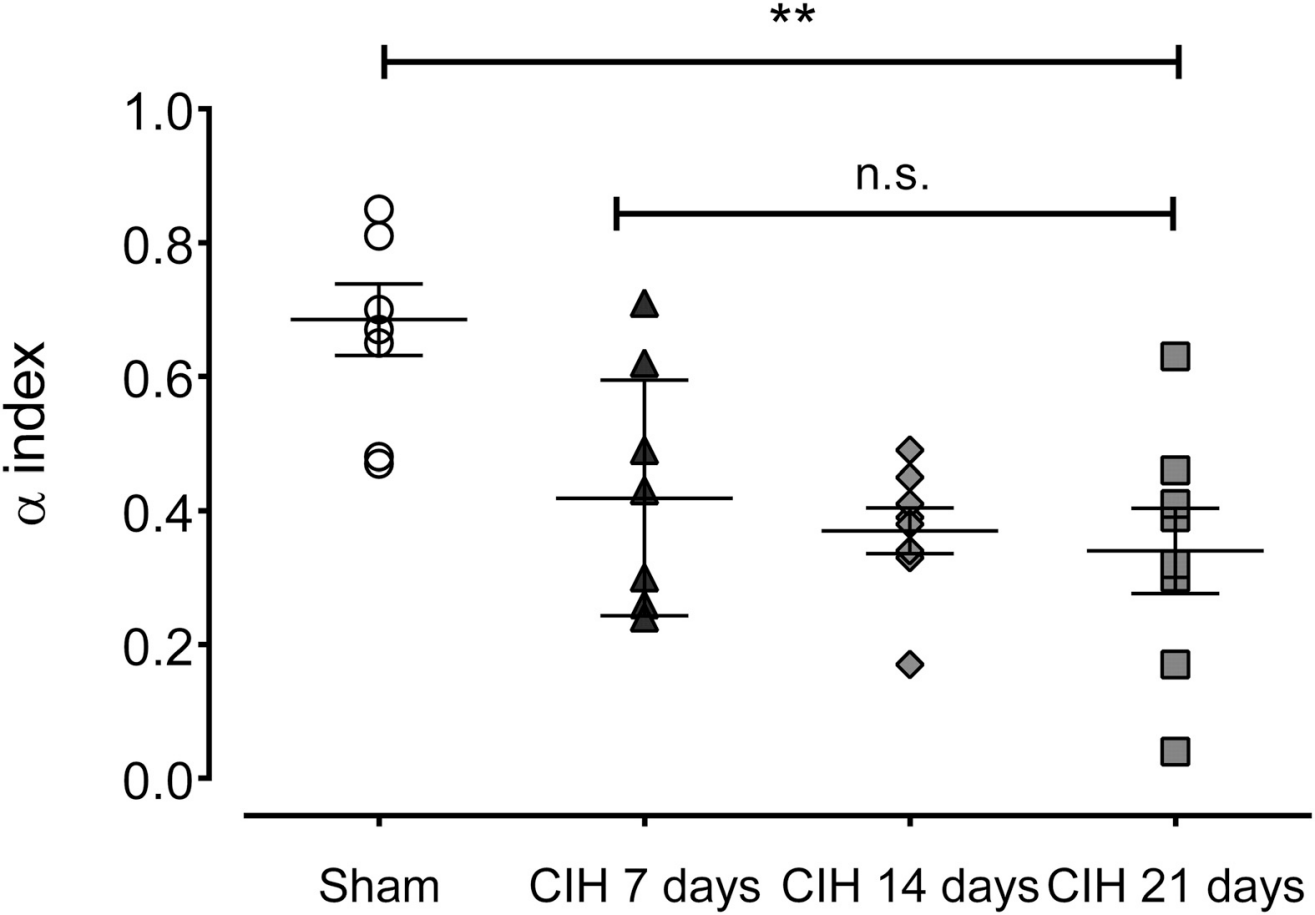
**Spontaneous baroreflex sensitivity after exposure to CIH for 7, 14 and 21 days, measured by spectral methods (α index)**. ^**^P < 0.01, n.s. *P* > 0.05., Newman-Keuls test after Two-Way ANOVA. *n* = 8–12 rats in each group.

**Figure 4:**
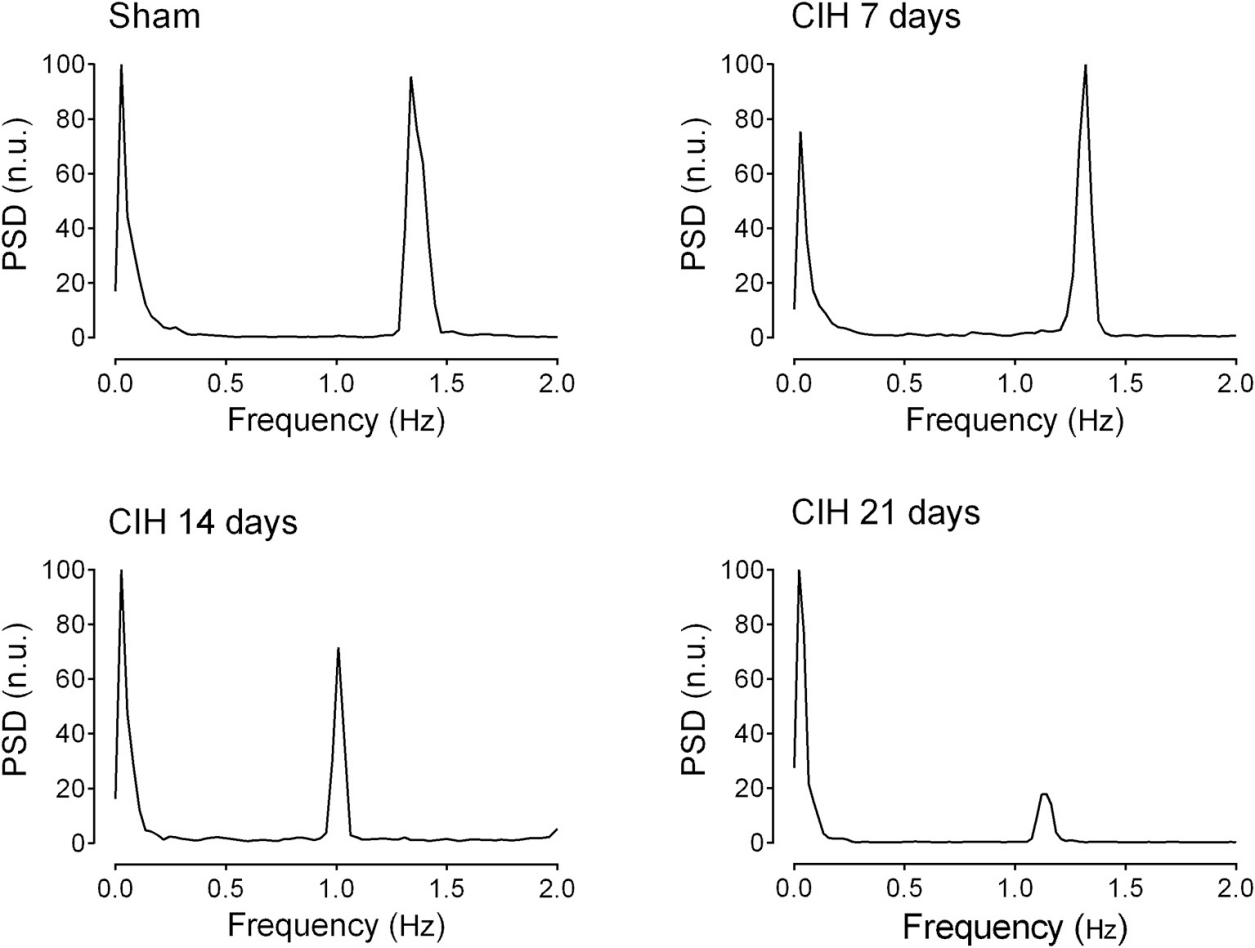
**Representative traces of the power spectral density of heart rate variability in one sham rat (Sham), one rat exposed to CIH for 7 days (CIH 7), one rat exposed to CIH for 14 days (CIH 14) and one rat exposed to CIH for 21 days (CIH 21)**. PSD, Power spectral density expressed in normalized units (n.u.).

**Figure 5:**
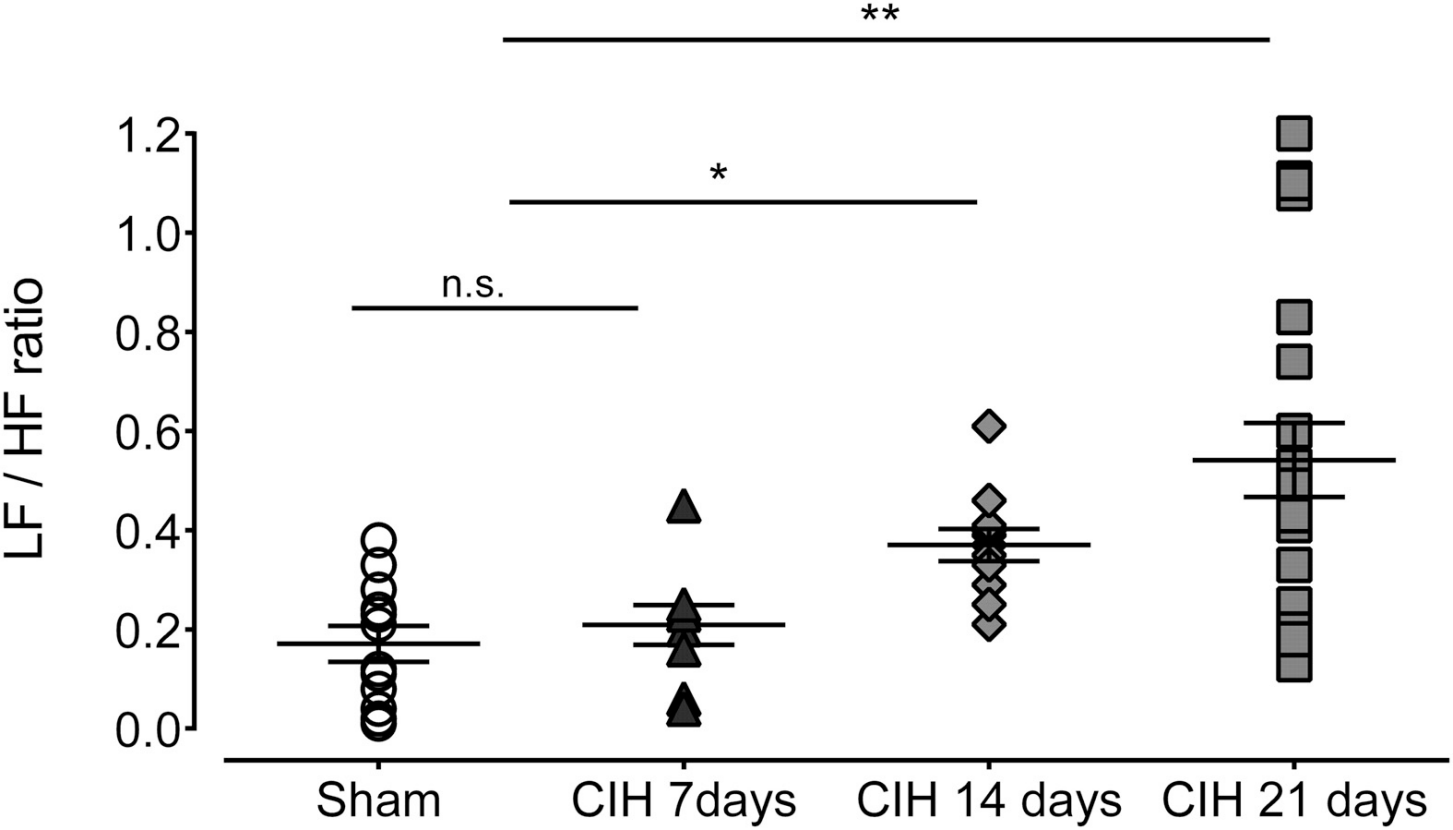
**Heart rate variability (HRV) in rats exposed to CIH for 7, 14, and 21 days. LF, low frequency band; HF, high frequency band**. ^**^P < 0.01, ^*^P < 0.05, n.s. *P* > 0.05, Newman-Keuls test after Two-Way ANOVA. *n* = 8–12 rats in each group.

Baroreflex sensitivity was significantly impaired by CIH. Indeed, CIH-treated rats displayed a reduce BRS compared to Sham animals (Figure 3). We found that CIH-treated rats after 7 days display a marked reduction in the BRS compared to the values obtained in sham rats and that BRS values remained reduced until the day 21 of exposure to CIH (*P* < 0.01, One-Way ANOVA). Rats exposed to CIH showed autonomic dysfunction characterized by shifts in the HRV power spectrum. Figure 4 shows representative power spectrum analyses of R-R variability in one Sham rat and rats exposed to CIH for 7, 14, and 21 days, respectively. Indeed, rats exposed to 14 and 21 days to CIH displayed marked changes in the distribution of the relative spectral components of HRV with significantly higher LF/HF ratio (*P* < 0.05 One-Way ANOVA, *n* = 8–12) as compared to rats exposed to CIH for 7 days or Sham rats (Figure 5).

### Interactions between the ventilatory chemoreflex gain, baroreflex sentivity and heart rate variability following CIH

**Figure 6:**
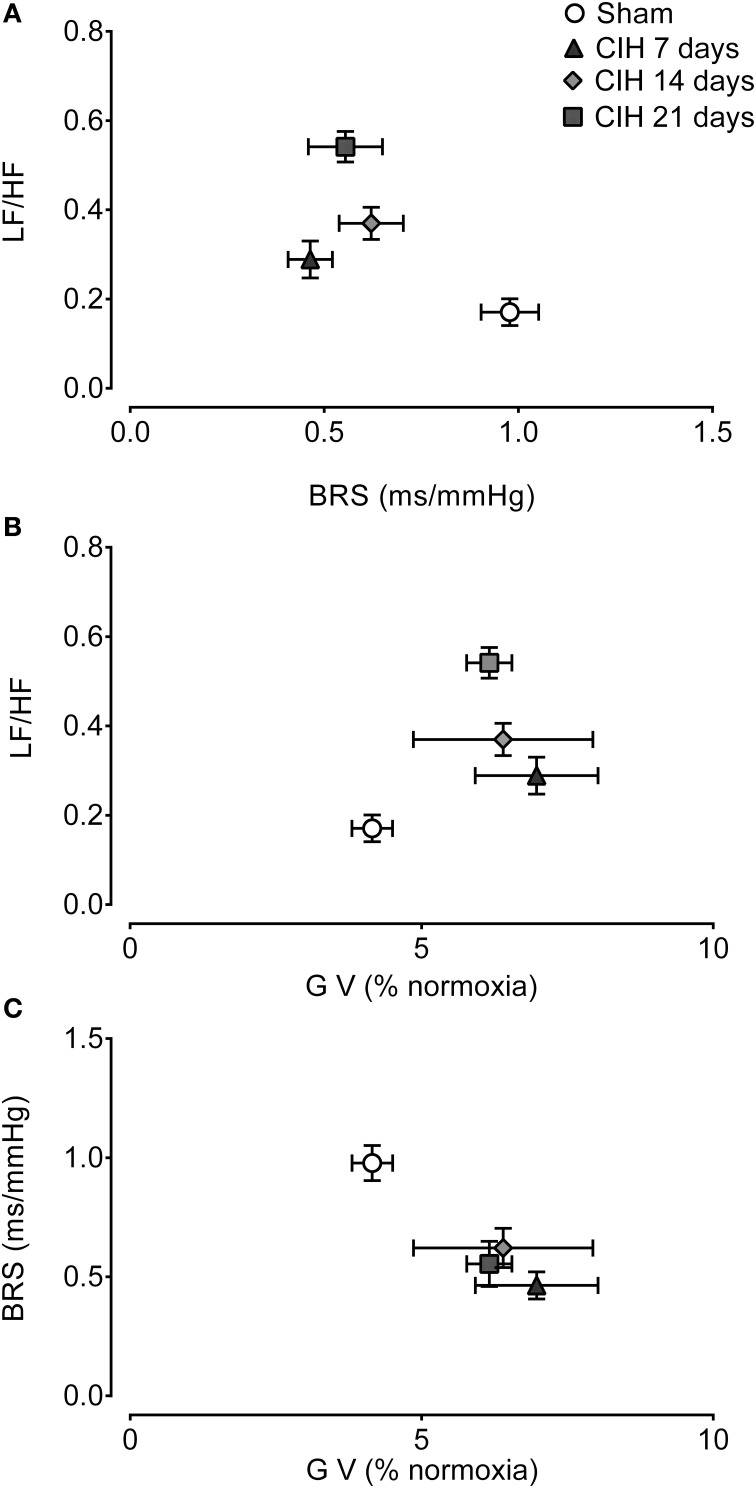
**Interactions between cardiorespiratory variables following CIH**. Interaction between spontaneous baroreflex sensitivity (BRS) and heart rate variability low to high frequency ratio (LF/HF) **(A)**, the gain of the ventilatory chemoreflex (GV) and LF/HF **(B)** and GV and BRS **(C)**. Data mean ± *SE*.

Figure 6A show the interaction between the spectral LF/HF index and BRS. The CIH-induced attenuation of BRS preceded the changes in the LF/HF spectral index of HRV. Indeed, BRS decreased after 7 days of CIH without a large change in the LF/HF ratio, but after 14 days the LH/HF index was significant higher. The co-variation of the gain of the CB-mediated ventilatory chemoreflex and LF/HF was characterized by a small increased in the ventilatory gain at 7 days of CIH exposure, followed by further increases of the LF/HF at 14 and 21 days of CIH (Figure 6B). Finally, the analysis of the BRS and the CB-mediated ventilatory chemoreflex gain showed that CIH induced a concomitant decrease of the BRS along with the increase in the ventilatory gain during the CIH exposure (Figure 6C).

## Discussion

Present results provide a coherent and integrative explanation of the role played by the enhanced CB chemosensory responses to hypoxia in the progression of the cardiorespiratory alterations and the hypertension induced by CIH, measured 18–24 h after the last intermittent hypoxic stimuli. The main findings of this study show that exposure of rats to cyclic episodes of CIH initially enhance the CB afferent chemosensory drive, decrease BRS and modified the HRV power spectrum toward a predominant sympathetic tone, and finally led to persistent hypertension. Thus, our results support the idea that the persistent hypertension induced by CIH is preceded by changes in CB chemosensory. The potentiation of CB chemosensory and reflex ventilatory responses to hypoxia, as well as the BRS and HRV alterations persisted when rats exhibited a significant elevation of BP in the absence of the CIH stimulus. The novel finding of the present study is that CIH induced a simultaneous decrease of BRS gain along with the potentiation of CB-mediated chemoreflex drive suggesting that the increases in the CB afferent activity may modify the sensitivity of the cardiac baroreflex being the final outcome the development of autonomic imbalance and high blood pressure during exposures to CIH.

### Role of the carotid body in the development of hypertension following CIH

A growing body of evidence supports the proposal that an abnormal enhanced CB chemoreceptor responsiveness to hypoxia is involved in the generation of the cardiorespiratory alterations and the hypertension in OSA patients and animals exposed to CIH. Patients with recently diagnosed OSA show enhanced ventilatory, sympathetic, and vasopressor responses to acute hypoxia, attributed to a potentiation of the peripheral chemoreflexes (Cistulli and Sullivan, 1994; Narkiewicz et al., 1998b, 1999). Indeed, Narkiewicz et al. (1999) studied the reflex ventilatory, tachycardic, and pressor responses to acute hypoxia in untreated normotensive patients with OSA, and found that hypoxic stimulation produces larger increases in minute ventilation, heart rate, and arterial blood pressure than control subjects. On the contrary, ventilatory, and pressor responses induced by hypercapnia and by the cold pressor test in OSA patients were not different than those observed in control subjects. In addition, Loredo et al. (2001) reported that OSA hypertensive patients present higher basal tidal volumes, suggesting an enhanced peripheral chemoreflex drive. Similarly, animals exposed to CIH show enhanced hypoxic ventilatory responses to acute hypoxia (Peng et al., 2003; Peng and Prabhakar, 2004; Rey et al., 2004; Iturriaga et al., 2009; Del Rio et al., 2010) and long-term facilitation of the respiratory motor responses (McGuire et al., 2003; Peng et al., 2003).

### Autonomic imbalance during CIH

Autonomic dysregulation has been proposed as a plausible mechanism involved in the progression of the hypertension in OSA patients and animals exposed to CIH. Cyclic hypoxic-reoxygenation episodes in OSA patients enhance the ventilatory, cardiovascular, and sympathetic responses induced by acute hypoxia (Carlson et al., 1993; Narkiewicz et al., 1998b). Similarly, animals exposed to CIH show augmented sympathetic outflow and develop systemic hypertension (Greenberg et al., 1999; Dick et al., 2007; Zoccal et al., 2009). In addition, the autonomic dysregulation has been associated with a reduction of the efficiency of the baroreflex control of heart rate and alterations of HRV in OSA patients (Shiomi et al., 1996; Narkiewicz et al., 1998a) and animals exposed to CIH (Rey et al., 2004, 2008; Lai et al., 2006; Sica and Zhao, 2006; Lin et al., 2007). Moreover, it has been shown that CIH produces parasympathetic withdrawal, which can further worsen the autonomic imbalance (Lin et al., 2007; Yan et al., 2008).

The spectral analysis of HRV has two major components defined as the low frequency (LF) band related to sympathetic influences, and the high frequency (HF) band related to the vagal influences and respiratory sinus arrhythmia. The LF/HF ratio is used as an index of the sympathovagal balance on heart rate (Task Force, 1996). Normotensive patients with recently diagnosed OSA showed a shift of the HRV spectral indexes toward the LF band, which is associated with increased sympathetic discharges in the peroneal nerve (Narkiewicz et al., 1998b). Lai et al. (2006) found that 5 days of CIH exposure increase the LF/HF ratio in awake rats. Our results in anesthetized rats exposed to CIH showed significant changes in HRV after 14 days of CIH, with a relative predominance of the low frequency component, suggesting a predominance of sympathetic modulation of heart rate. Interestingly, the time required to produce hypertension in rats exposed to CIH seems to be critically dependent on the hypoxic pattern, the severity, and the length of the exposure. Indeed, Fletcher et al. (1992) found an elevated BP in conscious rats after 7 days of CIH exposure, which was not different at 35 days of CIH exposure, indicating that the sustained increase in arterial pressure was maximal after 7 days of CIH. Similarly, Sica et al. (2000) and Peng and Prabhakar (2004) found that systolic arterial pressure increases in rats after 10 days of CIH. On the other hand, Allahdadi et al. (2008) using telemetric BP recordings found that MABP increased in rats after 14 days of exposure to a CIH pattern consisting in 90 s of N_2_ 100% followed by a 90 s of air, for 7 h per day. Our results showed that BP recordings obtained from anesthetized rats exposed to CIH display increases in systolic and diastolic BP after 21 days of CIH. Therefore, future studies are needed to address the effects of the use of several anesthetic agents on the cardiovascular alterations induced by CIH.

### Cardiac baroreflex alterations induced by CIH

Present results showed an attenuation of BRS, which preceded the changes in HRV and the hypertension. The changes in BRS found in this study suggest that the CIH-induced hypertension is associated with a reduction of the baroreflex gain. Some studies found that OSA produces a reduction in baroreflex sensitivity, which is associated with hypertension in humans (Carlson et al., 1996; Bonsignore et al., 2002) and animals exposed to OSA (Lai et al., 2006; Gu et al., 2007; Lin et al., 2007; Dematteis et al., 2008; Yan et al., 2008; Chalacheva et al., 2013). However, others studies performed in juvenile rats exposed to CIH for 10 days suggest that the hypertension is not secondary to the reduction in cardiac baroreflex, but to an enhanced respiratory–sympathetic coupling (Zoccal et al., 2009). Furthermore, Yamamoto et al. (2013) provide evidence that CIH for 7 days, which increases BP, may reset the renal efferent arm of the baroreflex control in rats without changes in the maximum gain. Moreover, Yamamoto et al. (2013) proposed that acute activation of peripheral chemoreceptor with hypoxic challenges could lead to baroreflex resetting. Here we found that CB chemosensory discharges are enhanced early during CIH exposure and that changes take place with a concomitant reduction in the BRS gain. Then, it is plausible that chemoreceptors and baroreceptors interact to establish autonomic dysregulation during CIH.

### Chemoreflex and baroreflex interaction: a link to CIH-induced hypertension

Chemoreflexes and baroreflexes play a main role in the control of the cardiorespiratory function in health and disease. Several studies reported interactions between peripheral chemoreceptors and arterial baroreceptors (Heistad et al., 1975; Mancia et al., 1976; Somers et al., 1991). It has been shown that hypoxic chemoreflex activation elicits baroreflex inhibition mainly attributed to central nervous system modulation at the level of the nucleus of the solitary tract (Miura and Reis, 1972). The available evidence suggests that an enhanced CB chemoreflex drive contributes to the cardiorespiratory alteration induced by CIH (Mitchell et al., 2001; McGuire et al., 2003; Peng et al., 2003; Peng and Prabhakar, 2004; Rey et al., 2004; Del Rio et al., 2010). Indeed, CIH enhanced CB chemoreceptor response to hypoxia (Peng and Prabhakar, 2004; Rey et al., 2004; Del Rio et al., 2012), which in turn produces sympathetic activation and hypertension. Accordingly, a blunted baroreflex response to the subsequent hypertension during CIH, may contribute to the further deterioration of the reflex control of BP. Indeed, we found that rats exposed to CIH displayed CB chemosensory potentiation and BRS reduction with the same time-course. Then, our results suggest that repetitive activation of the peripheral hypoxic chemoreflex during CIH result in alterations of the baroreflex control. Nevertheless, we cannot rule out that exposure to CIH *per se* may reduce BRS, or reset the baroreflex set-point to operate at a higher level of BP. Further studies are needed to prove that CB potentiation leads to baroreflex impairment following CIH.

### Study limitations

A major limitation of the present study is that experiments were performed under pentobarbital anesthesia. Unfortunately, this is an obligatory and necessary condition for recording carotid sinus nerve chemosensory discharges in spontaneously breathing animals. The fact that ECG and BP data were collected in anesthetized rats is a methodological limitation because anesthesia may influence HRV and BRS. Indeed, is well known that pentobarbitone anesthesia may induce autonomic depression (Yang et al., 1996) and reductions of the BRS (Shimokawa et al., 1998). We maintained a level of surgical anesthesia (stage III, plane 2) ascertained by the absence of withdrawal reflexes to strong pressure on the paws, with persistence of patellar reflexes. Accordingly, the level of anesthesia was the same to all the experimental groups used in our study. A future comprehensive study is needed to establish a comparison between conscious and anesthetized states.

## Conclusions

In summary, short-term CIH increases CB responsiveness to hypoxia, potentiates the reflex ventilatory response to hypoxia, reduces the baroreflex sensitivity and alters the spectral distribution of the HRV toward the sympathetic modulation, without an increase in BP. In addition, we found that CIH induced a simultaneous decrease in BRS gain along with an increase of the hypoxic reflex ventilatory gain, suggesting that the potentiation of peripheral chemoreflexes induced by CIH contributes to the autonomic imbalance. Following long-term CIH, the rats became hypertensive and still displayed enhanced CB chemosensory and reflex ventilatory responses to acute hypoxia, BRS impairment, and HRV imbalance. Together, our results show that CB afferent activity is potentiated early during the exposure to CIH and that precedes the onset of hypertension. Therefore, present results further support the role of peripheral carotid chemoreceptors in the context of CIH-induced autonomic dysregulation and hypertension.

### Conflict of interest statement

The authors declare that the research was conducted in the absence of any commercial or financial relationships that could be construed as a potential conflict of interest.
